# Onboarding: How Newcomers Integrate into an Agile Project Team

**DOI:** 10.1007/978-3-030-49392-9_2

**Published:** 2020-05-06

**Authors:** Peggy Gregory, Diane E. Strode, Raid AlQaisi, Helen Sharp, Leonor Barroca

**Affiliations:** 6 University of Oslo, Oslo, Norway; 7 Monash University, Clayton, VIC Australia; 8 IT University of Copenhagen, Copenhagen, Denmark; 9 University of British Columbia, Vancouver, BC Canada; 10 University of Central Lancashire, Preston, UK; 11 Whitireia Polytechnic, Wellington, New Zealand; 12 Independent Researcher, Glasgow, UK; 13 The Open University, Milton Keynes, UK

**Keywords:** Agile team onboarding, Onboarding, Newcomers, Scrum, Self-organizing team onboarding

## Abstract

Although a stable team is deemed optimal for agile project success, new team members need to join ongoing agile projects. Newcomers must rapidly assimilate into the organisational and project environment while learning how to contribute effectively to the project and integrate into the team without seriously interrupting project progress. This paper addresses how newcomers integrate into an established agile project team and the challenges newcomers and the team face during this process. This paper is a single case study of a co-located agile project team in a large IT department who regularly onboard inexperienced newcomers. We found a mixture of traditional onboarding practices and specific agile practices contribute to the onboarding process. Onboarding challenges include empowerment and mindset change, accommodating part-timers, conveying agile principles, and adjusting to changes in team composition.

## Introduction

Software development is a knowledge-intensive activity that relies on people with advanced technical knowledge, skills, experience, and domain knowledge. To organise software development, the commonly accepted approach is to adopt the mindset, process, and practices of agile software development. Agile software development is currently used in co-located, distributed, and large-scale systems development projects [1, 2], and within these environments, agile development optimally occurs in self-organising teams that are autonomous, cross-functional, and self-improving [3]. Newcomers to these environments face challenges in becoming fully integrated and productive team members. The challenges involve acquiring organisational knowledge, project knowledge, product and domain knowledge, knowledge of the technical environment, as well as understanding and becoming proficient in the agile approach used by the team, and undergoing socialization into a self-organising team environment [4].

Onboarding is the term used to describe new employees joining and integrating into an organisation. There is extensive literature on onboarding in organisations extending back to the 1970s [5, 6], and significant research into onboarding in Open Source Software Development projects [7, 8], as well as literature on onboarding in software development organisations [9]. There is a dearth of research into onboarding into co-located agile software development project teams with a single paper indicating that certain agile practices contribute to onboarding [4]. Onboarding was raised as a concern by practitioners in an international research-practitioner workshop in 2019 [10]. Practitioners are also concerned with sustaining successful agile project teams [11], and integrating newcomers is a factor in achieving long-term sustainability.

We expect onboarding into agile project teams will be similar in some respects to organisational onboarding in general, but also different to traditional onboarding because of the need for newcomers to understand the agile mindset, process, and practices and to effectively integrate into projects where self-organising teamwork is the norm. Therefore, we sought to understand the onboarding experiences of newcomers and their colleagues, into an ongoing co-located agile software development project team, how newcomers are integrated and how they learn the unique agile approach of the team. This study addresses the question: *How do newcomers integrate into an ongoing agile project team and learn the agile approach?* To address this question, we undertook a single case study of a co-located agile project team in a large IT department who regularly onboard inexperienced newcomers. We found a mixture of traditional onboarding practices and specific agile practices contribute to the onboarding process and several challenges occur for newcomers and established team members.

This paper is organised as follows. We first review pertinent literature on onboarding and describe Bauer’s framework [12], which we used to frame our analysis. Our case study method is described followed by our findings. The findings include a description of the agile project team, an analysis of the onboarding practices both agile-related and traditional, and an analysis of the key issues in onboarding for this team. A discussion of our contributions follows with a conclusion that includes ideas for future work.

## Background

*“Organizational socialization, or onboarding, is a process through which new employees move from being organizational outsiders to becoming organizational insiders. Onboarding refers to the process that helps new employees learn the knowledge, skills, and behaviors they need to succeed in their new organizations*” [5, p. 51].

In onboarding, a central idea is that of the *newcomer.* A newcomer is a new staff member joining an organisation. Newcomers also include people moving within the organisation, for example from one department to another or from one team to another. These people are organisational insiders, although not yet team insiders.

Onboarding literature emerged in the field of organisation studies in the 1970s when Maanen and Schein [6] defined the concepts of organisational socialisation, newcomers, insiders, and outsiders. Their idea was that organisations have functional, hierarchical, and inclusionary boundaries that newcomers cross as they are socialized from being outsiders to become insiders. Socialisation has six inter-related dimensions [6, p. 37] (the comments in brackets are our explanations).

1. Collective vs. individual socialization processes (join as a group or individually)
2. Formal vs. informal socialization processes (formal training or experiential learning)
3. Sequential vs. random steps in the socialization process (formal hierarchy of achievements or ad hoc, ambiguous achievement requirements)
4. Fixed vs. variable socialization processes (timetabled steps or no scheduled steps)
5. Serial vs. disjunctive socialization processes (role models or no role models)
6. Investiture vs. divestiture socialization processes (build on a person’s skills, values, attitudes or rebuild the person to fit the organisation)

Onboarding in commercial software development organisations was studied by Sharma and Stol [9]. After a review of empirical studies, these authors found nine studies of onboarding in software development organisations. They developed and tested a theoretical model of the relationship between onboarding activities (orientation, training, and support), onboarding success, organisational fit (job satisfaction and workplace relationship quality) and turnover intention. One key result was that orientation and support are strongly related to onboarding success.

Britto, Cruzes, Smite and Sablis [13] report a study of onboarding in three cases of globally distributed legacy software development, using the onboarding framework of Bauer [12] (described below). One key finding was that the greatest challenge was onboarding remote developers to an ongoing project when agile methods were followed because of the minimal documentation and the need for continuous dialogue with mentors to understand the project.

Onboarding in co-located agile project teams is addressed by Buchan, MacDonell, and Yang [4]. From an initial systematic literature survey, they identified 11 goals in the general software development literature that they determined were also relevant for agile onboarding (adapted from [4, p.3]).

- Understand and fit with company culture
- Understand and fit with team norms
- Understand and meet others’ expectations and one’s own role’s responsibilities.
- Understand the responsibilities, expertise and authority of other team members
- Understand what work to do and when
- Understand how to code and test to the team’s expectations
- Understand the team’s standards of team quality
- Understand and adopt the agile mindset
- Know how to use agile artefacts and techniques used by the rest of the team
- Understand the short, medium and long-term work structures, aims and implications
- Understand the product/project domain knowledge and terminology

This research reported 24 techniques for onboarding [4] and found that, among many traditional onboarding techniques such as access to formal training and access to online communities, the following agile practices contributed to onboarding: simple task, pair programming, retrospectives, and stand-up meetings. Due to the small number of research participants, 11 interviews in different organisations in New Zealand, these researchers acknowledged their list of onboarding techniques is unlikely to be exhaustive.

### Bauer’s Onboarding Framework

To frame the onboarding processes discussed in this paper we used the six functions described in Bauer’s framework for successful onboarding [5, 12]. Bauer’s framework is generic to all onboarding environments and situations. We selected this framework to structure our study because it is empirically based, highly cited in many fields, and currently no substantial framework or model exists for agile software development project team onboarding. The six functions in Bauer’s framework [12] are as follows:

- ***Recruiting process*** – The process that provides information to newcomers and helps them form realistic expectations of the organisation and their role. The recruiting process can be separate from the onboarding process but has been shown to be more effective if integrated into onboarding.
- ***Orientation*** – The process of helping newcomers to understand the important aspects of their jobs and of the organisation including the organisation’s culture, values, goals, history, and power structure. Orientation includes formal face-to-face, written guidelines, and online programmes for providing key information to newcomers. Orientation includes socialization, which involves making newcomers feel welcome by introducing them to co-workers and other people in the organisation.
- ***Support tools and processes*** – Support tools include a written onboarding plan for newcomers that includes timelines, goals, responsibilities, support systems, and how to access assistance. Attending regular meetings with a variety of stakeholders within the organisation is a mechanism for support of newcomers. Online support tools are another mechanism for onboarding but have been shown to be somewhat less effective than regular face-to-face orientation sessions.
- ***Coaching and support*** – Coaching, mentoring, and having role models are mechanisms for helping newcomers learn about the organisation and their role, and to navigate the social and political aspects of the organisation. Coaching and mentoring can be external or internally sourced. Using mentors is shown to improve newcomer knowledge of the organisation.
- ***Training*** **–** Training includes learning hard, soft, and onboarding skills. Training can be informal (learning-on-the-job) or formal (mandatory scheduled courses).
- ***Feedback tools*** **–** Feedback and guidance provide newcomers with information on progress, strengths, and weaknesses. Feedback can be formal (e.g. performance appraisals) and informal (e.g. the newcomer is proactive in asking questions about the expectations and evaluations of co-workers and supervisors).

Bauer’s [12] framework also includes adjustments that newcomers move through during onboarding. These adjustments are self-efficacy, role clarity, social integration, and knowledge of the culture. We have restricted our study to the six features in the framework because evaluating adjustments requires longitudinal research.

## Method

An organisation approached our research group and asked for assistance in identifying how to help new team members shift from an individual view of working to a team-oriented view of working when they joined an agile team for the first time. A single case study was selected as an appropriate method for addressing the research question with the unit of analysis being the co-located agile software development team [14]. The University of Central Lancashire gave ethical approval for the research.

The data was collected primarily by interviews. All people in the project team were asked if they would agree to be interviewed and were provided with an information sheet about the research. More than half of the project team were interviewed. The set of interviews covers a range of newcomers – new hires and those who had worked for up to a year in the project team – and insiders – established team members who had worked for 1 year or more in the project team and included the Team Lead/Scrum master – who had the longest experience in the project team.

Initial meetings and observations occurred in October 2018 followed by interviews and observations of the workplace in November and December 2018. Two researchers carried out the interviews. The interviews were semi-structured and followed an interview schedule, but the interviewers strived to remain open to new ideas and probed for additional information when necessary or relevant to the topic. All interviews were transcribed, and then analysed using the NVIVO tool. Table 1 shows the profile of the interviewees.

**Table 1.** Profile of interviewees

	Role and code name	Work Mode	Duration in role	Experience
1	Team lead; Scrum master [TL]	FT Perm	10 years	Degree; Certified Scrum Master Prior development and agile experience
2	Assistant project manager [PM]	FT Perm	6 years	Degree 4.5 yr as a student then employed FT No prior agile experience
3	Software developer 1 [SD1]	FT Perm	5 years	Degree 4 yr as a student then employed FT No prior agile experience
4	Software developer 2 [SD2]	FT Perm	1 year	Degree 3.5 years prior development experience Prior agile experience
5	Software developer 3 [SD3]	FT Perm	1 year	Degree Prior development experience prior agile experience
6	Apprentice developer [NC1]	PT Temp	8 months	Studying No prior development experience No prior agile experience
7	Student developer [NC2]	PT Temp	4 months	Studying Minimal prior development experience No agile experience
8	Software developer [NC3]	PT Temp	3 months	Degree prior agile and development experience 3 yrs at the university in another section
9	Conversation specialist [NC4]	PT Temp	3 months	Degree No prior development experience No prior agile experience

Observations of daily work and specific meetings were undertaken to get to know team members, observe how the team worked and aspects of team culture, and to identify problems. Observations were recorded with field notes during and immediately following the observation session.

The interview transcripts were initially coded by the first author for themes related to onboarding approaches, practices and challenges, following the coding guidelines of Saldana [15]. The data was also analysed to understand the team’s history, work practices (both social practices and agile practices), and the organisation and team culture. Once this was complete the first and second author mapped the onboarding approach and practice themes to the six functions in Bauer’s framework [12], described in Sect. 2.1. The second author then further analysed the themes to separate agile-related and traditional approaches. All authors reviewed the final analysis, and a draft of the paper was shared with the research participants for review and discussion before submission.

## Findings

### The History and Nature of the Agile Team

The agile software development team was a unit based in a UK university within the IT services section (ISS). Over six years, the unit increased from two members at inception to 15 at the time of the study. During the case study, the unit acted as a single team following a whole-team approach regardless of how many staff they had. The unit’s remit was to develop mobile applications for the university and investigate ideas and technology for future innovation. The unit worked on new projects and maintained deployed apps and systems.

Team membership and size changed depending on workload, consisting of full-time, part-time, experienced, inexperienced, student, and apprentice members. At the time of this study, there were 6 full-time staff, 3 apprentices and 6 part-time staff. Of the part-time staff 3 had full-time roles within the university and were part-time in this team. The team lead was full-time and had a duel role as Scrum Master and line manager. Apprentices worked full-time for most weeks but attended block courses, typically for one week per month, at their home institute. Student team members usually studied at the university while working part-time on the project team, typically for 2 or 3 mornings or afternoons per week. Most of the team were in their 20 s with little or no previous work experience except the Scrum Master, Product Owner and Conversation Specialist who were in the 35–55 age group and had a range of previous experience. Many of the full-time staff had started as part-time students and gained full-time posts as new graduates. There was regular staff turnover as students and apprentices left after graduating, and full-time staff were often attracted by jobs outside Higher Education.

The team developed their use of agile methods over time. In the early days, agile use was not systematic *“when I first started work, we were quite a small team, and we didn’t follow any methodology strictly, it was a bit ad hoc almost. We did follow the idea of sprints and some tokens of agile but not the sort of full beast that it is. It’s only once the team has grown that we have scaled up our utilisation of agile” [SD1].*

The team worked in an open-plan office space with an adjacent meeting room. The developers used a hot-desk system and often changed the configuration of their desks to suit themselves. The team used a Scrum approach, running two-week sprints, with the last Friday a non-Sprint day used to complete other work. The team had daily stand-ups, sprint planning, sprint refinement, sprint review, and retrospective meetings, product demos and used a Scrum wallboard. The Team Lead held weekly one-to-one meetings with staff if they wanted it. The team was functioning well. The general feeling among the team was stated by a staff member who had been with the team for a year, *“Personally, I love it. It’s very relaxed. It’s quite dynamic, the way we do things. It’s just a nice workplace” [SD1].*

### Onboarding Practices

The team’s onboarding practices are described in the following sections, organised according to Bauer’s framework [12]. Note that all names are pseudonyms.

#### **Recruiting Process**

The recruitment process was formal and standardized for all staff who join the organisation. The process is mandated by the organisation and requires a job description, person specification, and advertisement. The process differs for full-time (usually permanent) and part-time (usually temporary students and apprentices) newcomers. The recruitment of full-time staff is formal and requires a trained balanced panel of interviewers, applications are evaluated using a scoring mechanism, and applicants are interviewed using standard interview questions. All applicants are expected to show evidence of creativity, enthusiasm, and hard work. Experience and technical knowledge are expected of full-time applicants whereas for student and apprentice applicants this is not expected. Once hired, full-time members get an institutional induction. Both full-time and part-time members get a personal welcome from the Team Leader and are assigned a mentor. There is also a Scrum Coach to help newcomers.

*Long*-*term recruitment*: The unit had a long-term recruitment approach that involved hiring temporary students and apprentices who would work within the team as part-time employees whilst completing their studies. In some cases, these people would finish their degree and then become full-time permanent staff members on the project. This approach provided permanent staff who required minimal onboarding because they had a pre-existing good team fit, and understood the organisation, the unit’s goals, products, technologies, stakeholders, and the teams’ agile approach.

*Onboarding during recruitment:* During recruitment interviews, newcomer’s knowledge gaps began to be identified. “One *of the things I do is in the interviews when we take people on, I try to understand what their understanding of agile is, to see how much of a gap there is …” [TL].*

#### **Orientation**

*“New staff” pack:* This document described things that new employees need to know and was given to all newcomers. This was described by one team member, *“Here’s everything you need to know about the team,” [PM].* The lack of detail about the team’s approach to agile was acknowledged as a missing element “… *there’s no formal element. There should be. I’ll hold my hands up and go, there should be.” [PM].*

*“How our team works” pack*: This document is given to newcomers. The document describes the project team members and explains what newcomers need to sign up to, how to get into TFS (Team Foundation Server™) and explains how the team works.

*“How our team works with the client” pack:* This document is sent to clients before they work with the team. The document explains how the team writes user stories, what client communication the team expects, and how the team tests and signs-off products. This document is also given to newcomers to provide an overview of team practices.

*Agile method pack*: The Team Lead informed the newcomers about agile practices by sending them a guide, *“New team members, I now send them a guide, the principles behind it. A Scrum Guide. I talk about the fact that this is what they do” [TL].*

*Socialising:* The project team made efforts to socialise with and get to know one another because they found this helped newcomers to trust the team and be more confident in interacting and communicating with one another. *“For example, practices that we’ve encouraged in our full*-*time team meeting, we’ll say ‘what can you present to the team that you think is valuable or about yourself?’, you know breaking down those barriers, it could be about anything. So [a team member] recently did one about e*-*capture and [another team member] did one about his passion for Rubik’s Cubes” [TL].* The whole team was invited to social events, *“the different team members will often be going for lunch, that sort of thing. The odd evening here or there, we’d be going for drinks in town or we do our own team Christmas thing…” [SD3].*

#### **Support Tools and Processes**

*Information radiator*: The project team used a Scrum/Kanban wallboard with physical and virtual versions, although they tended to prefer the physical board. The established members saw the physical board as useful also for newcomers, *“Sometimes the team don’t necessarily engage quite as much with a digital thing as with a physical thing, it seems to be a bit more natural…I think it helps [the newcomers] as well because it’s a more instantaneous way to look and see where things are.”[SD1].* The wallboard was viewed by one newcomer as useful for developers but not for him as an architect, *“it’s all development tasks that are on the board… now. But then, my work is stuff that just supports all of that, and sometimes it’s like, I want to write a story that is… ‘As an architect, I want’”* [NC3].

*Communication tools:* The team used communication tools including Teams, Slack, TFS, and email. These tools helped the part-time newcomers to some extent, although there was often quite a lot of missing information to catch up on during an absence, so part-timers also walked around the room to talk to people.

#### **Coaching and Support**

*Mentoring*: Mentoring was viewed as an important part of the onboarding experience for most newcomers. The Team Lead was frequently mentioned as a mentor but he also recognised the mentoring role of the established team members, “*from my perspective the mentoring aspect of things, it helps both with the integration into the unit, the integration with the technology stack and the integration into the agile way, and it’s kind of almost subliminal. The messages come across from the team members rather than from me, which, I hope, [the newcomer] would learn better because of that” [TL].*

*Role modelling*: The more experienced team members noted that role modelling desired behaviours was beneficial for the newcomer and the established staff, “*I try and get rid of the stigma … and set an example, and the rest of the team will realise that it’s fine to say ‘I don’t know how to do that. I don’t know what this is or that is, or I need help with this’” [SD3].* Another type of role modelling was shown by the continuous self-learning of new technologies by the established staff, *“I do a lot of learning outside of work at the moment, especially with all the new stuff that we’re doing” [PM].*

*Ceremonies:* As part of the immersion approach, ceremonies were explained to newcomers the first time they attended. For example, just before the stand-up meeting, a newcomer would have the process explained so they knew what they were expected to do, “*they were very good at explaining everything they did, explaining why they had stand*-*ups in the morning, and explain the meetings, you know, before and the end of the sprints. They explained that before they happened*” *[NC4].*

*Encouraging teamwork:* The established team members encouraged knowledge sharing and helping behaviours among the team*, “everyone is very friendly, and ask if you want anything and yeah, you are encouraged to talk to people*.” *[NC3].* The level of trust between newcomers and established staff was perceived as good, *“There’s a lot of trust… especially with the student developers as well, there’s a lot of trust for them to do work, … once they’re part of the team, and they fit and work as part of the team, we trust them to do work”. “Everyone is very helpful, very friendly… it feels very inclusive, very inclusive, it’s not sort*-*of developers and non*-*developers” [NC3].*

*Encouraging learning*: One newcomer appreciated being encouraged to try new things, *“[The TL] is very good at encouraging you to take on more challenging things. … He’ll suggest, why doesn’t [Sally] do that, why don’t you do that [Sally]? Initially, I’ll go ohhh (shouting in confusion and panic!) and then… But in a good way, it is good to push your staff, isn’t it? It is good to learn new things and yeah. Yeah, it is good. Scary but good. Good scary” [NC3].*

*Empathy:* Because some established team members had previously been student members in the team, they could still recall their own experiences and this helped them to understand newcomers issues*, “I’d like to think anyway, that we treat the students, especially with my background as a student developer, that we’re all treated as equals. We don’t really have the junior developer syndrome that some teams suffer from where they’re handed lesser tasks or things like that. …Sometimes if a part*-*time student is only in for 3* *h or something, then there might be a situation where we might suggest things for them, just to maximise that time that they have. But it’s more for their benefit because I know how frustrating it is to get into a piece of work and then have to down tools and go to lectures” [SD1].*

*Pair programming:* The Team Lead recognised that pair programming was useful to support newcomers. *“When they first come in, I pair them up with a full*-*time member … the same full*-*time member for about 2 to 3 weeks until we then release them to work on their own on a particular area.” [TL].* Pairing was also used to learn new technologies, “*Where we want skills on a particular technology or something like that we’ll pair up, or equally if we want to teach someone something we’ll pair up” [SD1].*

*Reimagining yourself:* The Team Lead encouraged the newcomers to reimagine themselves in their new role, “*when I’ve taken students on and they’ve transitioned to being full*-*time members of staff, I’ve tried to coach them to say you need to reimagine yourself in the new role. So [newcomer]…, she was an administrator but now she’s a, well technically her title is [new role], but that’s actually different to what she does and she’s had to reimage herself in those new roles because she’s no longer doing the roles that she was doing earlier on” [TL].*

*Daily stand*-*up meetings:* These meetings were held sometimes twice a day for the benefit of the part-time staff. One developer, with one year of experience on the team, saw the stand-ups as useful for understanding the project status and as a time for getting help, *“If you’re stuck on something, don’t know how to do something or you’re just lost, then it’s a good place to air that and usually, somebody will, oh I’ll help you with that.” [SD3].* One newcomer noted that she did not yet understand the language*, “If I understood their language, then I would probably understand more” [NC3].* Established members also saw that stand-ups helped newcomers*. “A lot of the communication comes at the stand*-*up in the morning… We also have another, sometimes, in the afternoon if someone’s come in just to get them on board. So we might have two stand*-*ups” [SD2].*

*Co*-*location:* Co-location made asking for help and sharing knowledge easier, *“…look at this code, or something like that but also, just asking the person you’re sat next to… If you don’t know something, there’s a good chance the person next to you does” [SD3].* Co-location allowed for conversations to be overheard, which helped newcomers, *“It does [give a] general sense of what other people doing, even if it’s just overhearing them have, talking between themselves” [NC1].*

*Signalling:* To signal availability and issues the team had developed methods of communicating so members could understand who could be interrupted and who preferred to focus on their work, or if there was an important issue for the team to address. *“Sometimes members of the team will wear headphones when they’re really concentrating so you know to stay clear, or you just from intuition just by knowing each other…And I think we’re all accessible to part*-*time students as well”. “if it’s a particular barrier in terms of the project, then we have little red notices that go on the Kanban board…so that everyone knows there is a barrier and if anyone has a solution … we can discuss and try to break that barrier down” [SD1].*

#### **Training**

*Formal training:* No formal training was available for full-time members of staff due to budget restrictions. In addition, most of the project team were not able to attend Agile Conferences or other external events due to the heavy workload. No formal courses were mentioned, but students and apprentices already attended formal courses of study.

*Immersion:* *(or experiential learning)* Newcomers started working in the team from their first day and much of the learning and socialisation was accomplished by being a productive member of the team. For example, two of the established members described the process in a similar way as, *“Generally we try to let them get their hands into a piece of work, learn literally on the job, so we give them a sort of induction into what their sort of expectations are in the team, what they can do to get support and all that kind of stuff and just let them loose and fit right in” [PM].* A newcomer’s perception reinforced this, *“I was very much thrown in at the deep end, “Here are some meetings. Yeah, let’s go ahead with it,” and very much learning on a day*-*to*-*day basis with the team how they do it”. “it’s really largely practice, or very practical, with some explanations when necessary… before we went into the meeting and we were voting with our animal cards and things, that was explained to me before we went in, we do this, so… I got in there and wasn’t surprised by what happened” [NC3].*

*Self*-*study:* Newcomers who were not aware of agile methods were asked to read about it before starting with the team and were given links to online resources. *“In the interviews, we tend to ask them if they have any experience of agile, and if they say no, we say, ‘That’s fine, but we recommend you look into it’” [PM].* The existing project team expected newcomers to self-learn and would request them to do so, *“when we took him on, we said ‘you need to do some learning outside of work if you want to continue with the team’” [TL].* For some newcomers, the self-study was self-motivated*, “I did a lot of background work …I did lots of reading [about Alexa] on the internet… A couple of courses on Udemy …At home, I am doing Python and Excel, I am doing a course on Excel. And … I have just signed up for, … user stories” [NC4].*

#### **Feedback Tools**

*One*-*to*-*Ones*: Full-time members of the team had regular, often weekly, one-to-one meetings with the team lead. This gave team members a chance to receive guidance about technical issues and reflect on their work practice.

*Immediate feedback:* The team was able to provide face-to-face feedback, as a newcomer explained after the testing of her work, *“people do point things out, but in an ok way… but it is always nicely done”. [NC3].*

*Meetings:* Meetings were used to communicate university, department, and team knowledge and concerns. “*everyone gets to say something in there. That’s working quite well. It’s nice and relaxed. It’s breaking down some barriers. People are understanding people better, and new learning is coming into the team.” [TL]*

*Code reviews:* Code reviews were used for providing feedback, *“We do a group code review each week to see what we’ve been going over, to learn off each other. That meeting is primarily just for the programmers and the apprentices” [SD2].* A newcomer, who had not yet presented at a code review, thought the code reviews useful, *“At the moment I don’t quite understand everything. But it is useful because it can be quite scary to have a look at the [code], it makes it a bit more familiar” [NC1].*

*Testing:* Unit tests were viewed as a feedback mechanism and some test-driven development was used during pair programming to assist newcomers, *“We do try pair programming, especially with the students… so, when [Martin] started, we actually added some testing*-*driven development with him to introduce him to what we’re working on, how we work” [SD2].* A newcomer explained, *“which is really good, because that extra bit of testing is, and then I can see whether it does what I hoped it will do and if it works” [NC3].*

*Retrospectives*: Retrospectives were used to adjust and improve the agile process, the established team members viewed them as a valuable feedback tool. *“We do it at the retrospectives or we give feedback on how we did, what we liked, what we’d improve. So that’s more feedback as a team” [SD2].*

*Sprint review:* Feedback at the sprint review was concerned with the technical matters, *“Feedback’s generally kept back for the sprint review, so before a retro, we do a review session where we demo the build. Hopefully, it works and we can celebrate, or there will be some critique about the way it’s been implemented or the design choices, or that kind of stuff” [SD1].*

*Sprint refinements:* The team used these sessions to discuss and refine user stories before sprint planning sessions. “*We have Sprint refinements before we do a planning, where we go through each of the work items and ask a lot of questions” [SD3].*

*Small tasks:* Smaller tasks were given to part-time newcomers for practical reasons. “*We’ll give smaller tasks to the students because there’s just not enough time… if we’ve got a small user story, say, getting the next timetable event from an API, that’s something that we could see a student doing” [SD2].* Minor bug fixes were often an entry point for newcomers, “*I’ll have like a list of bugs that need fixing because generally, we don’t want to pull the full*-*timers out of sprint.” [PM].*

*Task allocation:* A mixture of self-selection and supervisor selection was used for task allocation. Considerations of expertise were a factor in allocating tasks. *“On the bigger tasks, sometimes [TL] will delegate who to do that … But usually, we just pick up the next task on the board. If there’s no task on the board, then we have to ask [the administrator] or [TL] to bring it in or liaise with the product owner…” [SD2].*

*Product demo:* Feedback on the product was given by Product Owners to the team, “*Other bits [of feedback] will be demos to the business. So, as developers, we try to talk to the actual product owners quite regularly” [SD1].*

The findings from the analysis are summarised in Table 2.

**Table 2.** Summary of findings

Onboarding	Traditional	Agile-related
Recruiting process	Follow legal recruitment requirements Long-term recruitment	Evaluate agile knowledge and give resources
Orientation	Provide new staff pack Provide teamwork pack Socialise with newcomers	Provide agile fundamentals pack
Support tools and processes	Introduce all communication tools	Introduce and use an information radiator
Coaching and support	Mentoring Role-modelling Encourage learning Empathy Reimaging	Ceremonies – explain just prior Encourage teamwork Pair programming Stand-ups Co-locate Signalling
Training	Offer formal courses, training, and conferences on relevant topics	Immersion from day one Self-study
Feedback tools	One-on-ones with senior staff Immediate feedback during immersion Meetings – encourage staff to speak Small tasks	Code reviews Testing Retrospectives Sprint review Sprint refinements Task allocation

### Onboarding Challenges for the Newcomers and the Agile Project Team

Onboarding challenged newcomers and established team members. Challenges identified in the analysis included empowerment, mindset change, accommodating part-timers, conveying agile principles, and adjusting to changes in team composition.

Empowerment: was a constant issue within the team. The Team Lead identified a difficulty with onboarding younger newcomers who had never worked in a self-organising empowered team. He thought they needed to be helped, *“when they’re just out of university and they’ve come from an academic background that doesn’t teach team work very well, doesn’t teach about empowerment … sometimes in conversations, they may turn to me in terms of a position of authority and I’m like, no you go and do that, so I’ve tried to set up things where they have their own meetings and they run their own meetings so I may well initiate something and step out and say well there you go, you don’t need to talk to me anymore, just sort it out yourselves.” [TL].* However, at times of pressure, a command-and-control approach did emerge, *“and then I’ll pull someone out of sprint and go, “This needs fixing,” or I’ll say, “This will be fixed at the end of the sprint, depending on how urgent it is” [PM].*

Mindset change: Project team members tended to rely on senior staff to maintain their agile processes, *“If me or [the TL] aren’t in the office, stand*-*ups don’t happen, and so we’re really trying to encourage, ‘This is your meeting, this is for you to help each other’” [PM].* A constant effort was made to empower newcomers, *“We try to leave it down to them what they want to do” [SD1].* Related to the issue with empowerment, is the problem of perfectionism. Newcomers found it hard to adopt an experimental mindset, *“Because they’re so new, they also don’t understand how to tackle problems. It’s a case of, ‘well just start, just get started it doesn’t matter if you throw it all away’ … it’s a mindset thing about trying to find the perfect solution the first time you do it” [TL].*

Accommodating part-timers: A recurrent theme among the team was connecting with, and sharing knowledge with, the part-time newcomers. Both established members and part-time newcomers saw this as an issue, *“…for part*-*time members or [those] who can’t attend, and it’s probably trying to find ways of bridging the gap in the communications that occur. So, it’s kind of every time we’ve had a retro everyone has said, communications need to improve. It’s like you’ve said it, but you’re not actually doing it.” [TL].* A part-time newcomer commented on the difficulty of finding out what had happened in the project after an absence, *“because they’ll just talk to each other and just figure something out, and then you won’t find it documented anywhere, or it won’t even be in the [TFS]” [NC3].*

Conveying agile principles: The established members had high expectations of newcomers and struggled to convey agile principles. *“And we now have a very high bar of workforce that are now the team, are highly motivated and through that, there’s an expectation that you have to fit into that kind of ethos as well, and that becomes a barrier for recruiting new students because the bar is so high.” [TL].* The Team Lead thought the main onboarding issue was integrating relatively young and inexperienced part-time newcomers, “*It’s just them being immersed in it, and for part*-*time that’s hard, because up to 15* *h a week, whilst doing other learning, and whilst you’re young and having a social life, and everything else. Finding space in their brain for this is hard, and it’s being able to get over the principles and culture, which is what I want to focus on” [TL].*

Adjusting team composition: Over time the team evolved to consist of more established members and fewer newcomers. This balance improved their ability to continuously improve. *“We’ve been through a lot of iterations of how we approach our work, and I think we’re hitting a sweet spot of getting things done, …with having more full*-*time members we thought there was value in doing those [sprint refinements]” [SD1].*

## Discussion

This study explored the onboarding of newcomers into a co-located agile software development project team because of its interest to practitioners who want to sustain their teams over the long term. We addressed the question of how newcomers integrate into ongoing teams and learn the agile approach. Analysing our single case study using Bauer’s onboarding framework [12], we found that onboarding combines traditional and agile-related techniques (see Table 2). Agile-related techniques include self-study of agile fundamentals, information radiators, introducing ceremonies prior to experiencing the ceremony, pair programming, immersion for experiential learning, code reviews, testing, retrospectives, sprint reviews, sprint refinement sessions, and flexible task allocation. In our case, we also found onboarding issues. The issues included supporting newcomers to act in an empowered agile manner and approach the work with an experimental mindset, being flexible to support inclusiveness of part-time staff, that conveying agile principles is a challenge, and the proportion of established to newcomer staff affects continuous improvement.

Our findings support those of [4, 9], and [13], however, ours are based on an in-depth contextual study of onboarding practices in an agile team and provide more nuance than those prior studies. We identify additional agile practices that support onboarding and show the extensive use of coaching and feedback processes in agile onboarding. In addition, our study identifies specific onboarding challenges for newcomers and teams. The challenge not identified in these earlier studies is empowerment, more specifically, how to encourage newcomers to act in an empowered way.

Our study contributes to practice by providing guidance for agile project teams who want to better understand the role of specific agile practices in supporting onboarding, and which traditional onboarding techniques to use alongside these agile practices to provide comprehensive onboarding support. We provide three recommendations for agile practitioners 1) incorporate the agile-relate practices shown in Table 2 that support onboarding, 2) use a long-term recruitment approach such as hiring placement students and apprentices and hire from this pool to ensure good staff ‘fit’, and 3) focus on training, explaining, and modelling empowerment when onboarding staff.

For theory, our study supports traditional onboarding knowledge, as it is an example of the use of Bauer’s framework, and extends that framework to, at least partially, account for onboarding in co-located agile software development project teams.

Our study has limitations. Our findings are based on a single case study with a limited number of interviews, and we acknowledge our findings are of limited transferability to other settings. In addition, we did not interview the whole team, so some perceptions are missing. We did get insights from a range of people, from very new staff, staff with 1 year of experience, to long-established staff. Thus we achieved some triangulation of data sources [16]. We also carried out a member check by providing a report to the project team summarising our findings and asking for confirmation and feedback.

## Conclusion

In this paper, we claimed that onboarding newcomers to co-located agile software development projects might differ from onboarding in general. We found traditional onboarding practices are used in agile project teams and that certain agile practices taught using immersive learning also support onboarding. We also identified challenges in onboarding to an agile project team.

This paper makes three contributions 1) provides in-depth insights into onboarding in an established co-located agile project team and specifies agile and other practices that support onboarding including challenges faced, 2) shows that Bauer’s [12] onboarding framework is appropriate in a software engineering context, and 3) provides recommendations for practitioners as to those agile practices that support onboarding.

In future work, we recommend research to develop a comprehensive onboarding model that fully elaborates the factors in agile onboarding. That research should encompass onboarding in all agile environments, co-located, distributed and large-scale.
